# Editorial: PET/CT and MRI in prostate cancer

**DOI:** 10.3389/fonc.2024.1421542

**Published:** 2024-05-24

**Authors:** Fabio Grizzi, Gianluigi Taverna

**Affiliations:** ^1^ Department of Immunology and Inflammation, IRCCS Humanitas Research Hospital, Milan, Italy; ^2^ Department of Biomedical Sciences, Humanitas University, Milan, Italy; ^3^ Department of Urology, Humanitas Mater Domini, Castellanza, Varese, Italy

**Keywords:** prostate cancer, diagnosis, PET/CT, MRI, artificial intelligence

Prostate cancer (PCa) represents one of the leading causes of cancer-related mortality (1). Age, African ancestry, and a family history of PCa are widely recognized as established risk factors (2). PCa exhibits a wide spectrum of aggressiveness, ranging from slow-growing to highly life-threatening. Large-scale trials have demonstrated that low-grade PCa (grade group 1) is associated with a very low risk of cancer-specific death. On the other hand, cancers classified in grade groups 3 through 5 display significantly higher metastatic potential and accounted for the majority of the estimated deaths from PCa (3). This diversity in the lethality of different PCa subtypes underscores the critical need for precise and accurate diagnosis of PCa. At diagnosis, 13% of PCa patients will have regional lymph node involvement and 8% will have distant metastasis (4). The most common site of metastatic PCa (mPCa) involvement is the bone, accounting for up to 90% of mPCa. Visceral organ involvement, such as in the lung, liver, adrenal gland, and brain, is less common. When compared to localized PCa, the 5-year survival rate of mPCa declines significantly from 100% to 34.1%. Early detection of mPCa is crucial for treatment. The U.S. Food and Drug Administration (FDA) sanctioned the assessment of prostate-specific antigen (PSA), a protein discharged by both healthy and cancerous prostate cells, in 1986 (5). Initially authorized for tracking patients with confirmed PCa, it was later endorsed in 1994 to assist in detecting PCa alongside digital rectal examination (DRE) in individuals aged 50 and above. Recently, screening for PCa using serum PSA has come under considerable criticism due to several trials demonstrating that using PSA serum levels often leads to overdiagnosis and overtreatment, as well as the inability to accurately differentiate between low-, intermediate-, and high-risk aggressive disease. PCa diagnosis is currently based on the gold standard invasive procedure of transrectal ultrasound (TRUS)-guided needle biopsy of the prostate. The diagnostic biopsy is informed by the combination of any of the following: elevated PSA serum levels, PSA kinetics, abnormal DRE, family history, race, or abnormal previous biopsy. Gleason score, cancer stage, and cancer core information are all obtained from biopsy, and frequent or periodic biopsies are not amenable for patients. Individuals with elevated PSA levels upon screening have the option to pursue additional examinations to determine the necessity for biopsy, multiparametric magnetic resonance imaging (MRI) to pinpoint biopsy sites, or both. Those diagnosed with low-risk or favorable intermediate-risk PCa may opt for active surveillance, involving periodic PSA tests and biopsies, instead of immediate curative treatments such as surgery or radiation therapy (5). Using a 12-core systematic prostate biopsy tends to yield inaccuracies in diagnosis, leading to both overdiagnosis and underdiagnosis of prostate cancer (3). Employing MRI targeting during biopsies can potentially mitigate the misclassification of PCa, especially in men with MRI-visible lesions. In patients displaying MRI-visible lesions, utilizing a combined biopsy approach resulted in increased detection of PCa. Nevertheless, relying solely on MRI-targeted biopsy led to an underestimation of the histologic grade for certain tumors. Following radical prostatectomy, the occurrence of upgrades to grade group 3 or higher during histopathological analysis was notably reduced after implementing combined biopsy techniques (3). The European Association of Nuclear Medicine (EANM) has recently introduced a molecular imaging TNM (miTNM) classification utilizing prostate-specific membrane antigen (PSMA) positron emission tomography (PET) scan/computed tomography (CT) observations (6). It is anticipated that the prognosis of the miT, miN, and miM substages will likely be more favorable compared to their conventional T, N, and M counterparts due to the enhanced sensitivity of PSMA PET/CT over standard bone scans and abdominopelvic CT scans. However, the extent of this prognostic improvement and its practical significance and implications remain to be thoroughly evaluated. MRI of the prostate has been, however, recommended as the initial diagnostic test for men presenting with suspected PCa, with a negative MRI enabling safe avoidance of biopsy and a positive result enabling MRI-directed sampling of lesions (7). Evidence supports the role of the MRI-directed pathway for PCa diagnosis, with improved performance over the previous clinical standard of systematic TRUS needle biopsy of the prostate. In terms of localizing the primary tumor for diagnostic biopsy, MRI prior to biopsy is becoming common practice to identify more clinically significant PCa (International Society of Urological Pathology [ISUP] grade group ≥2) and reduce the diagnosis of non-clinically significant disease (8). The main role of prostate MRI is to detect only clinically significant PCa. The prevalence of clinically significant PCa in men referred to urology clinics has been reported as ~30%, indicating that a substantial proportion of patients might unnecessarily undergo an invasive biopsy procedure; however, a negative MRI would enable up to half of these patients to safely avoid biopsy (9). Conversely, a positive MRI can directly target tumor lesions to provide pathologically accurate tissue sampling (9). The negative predictive value (NPV) of MRI is high (~90%) and has little variability among centers, whereas a comparatively low positive predictive value (PPV) of 17%, 46%, and 75% has been reported for lesions with a Prostate Imaging- Reporting and Data System (PI-RADS) score of 3, 4, and 5, respectively (9). MRI lesions are assessed using the PI-RADS score, ranging from 1 to 5. Higher scores signify lesions that are more clinically suspicious, aiding in the stratification of PCa risk. Prostate MRI, when interpreted using the PI-RADS, enhances the initial detection of clinically significant PCa (csPCa) compared to standard biopsy, thus aiding in the reduction of overdiagnosis. However, despite these benefits, approximately 15% of csPCa cases may still evade detection. Additionally, the PPV of PI-RADS can vary among different institutions. To tackle these challenges effectively, strategies must be implemented to minimize interobserver variability in interpretation. Recognizing the evolving demands in prostate MRI interpretation, specialized scoring systems have emerged beyond PI-RADS to address specific scenarios and unmet needs. Examples include the Prostate Imaging Quality (PI-QUAL) score, designed for assessing the image quality of mpMRI examinations. Additionally, the Prostate Cancer Radiologic Estimation of Change in Sequential Evaluation (PRECISE) recommendations offer guidance for evaluating serial mpMRI examinations during active surveillance. For assessing local recurrence after radical prostatectomy or radiation therapy, the Prostate Imaging for Recurrence Reporting System (PI-RR) score is utilized, while the Prostate Imaging after Focal Ablation (PI-FAB) score is employed to assess local recurrence after focal therapy. These specialized scoring systems cater to specific clinical scenarios, providing tailored and comprehensive evaluation methods beyond the scope of traditional PI-RADS (10). It has been shown that MRI performs best as a rule- out test; however, results from studies in which MRI-detected lesions were compared with histopathology on prostatectomy specimens showed that 8–24% of Grade Group 2 PCa might be MRI occult (9), which could mainly be ascribed to technical limitations, the presence of cribriform glands, and/or a sparse pattern of tumor growth. It has been reported that the diagnostic strategy involving PSAs low sensitivity, the invasiveness of prostate biopsy sampling, and the variability in performing and interpreting MRI is constrained by various factors. Successful implementation of this approach necessitates experienced clinicians, optimized equipment, effective interdisciplinary communication, and standardized workflows. Each component of the pathway must be carefully executed to achieve the anticipated results. PCa can vary tremendously in its clinical behavior and response to treatment. Due to this and its substantial global incidence, there is an ongoing need for improved diagnostic, risk-stratification, and therapeutic approaches to optimize patient outcomes. PSMA PET has begun to revolutionize the landscape of PCa management from both a diagnostic and therapeutic perspective. PSMA, a transmembrane glycoprotein (11), was initially identified on prostate cells in 1987 (12) and cloned and characterized in 1993 (13). It was further noted to be preferentially expressed on malignant versus benign prostate cells, prompting researchers to develop it as a target for molecular imaging and theranostic applications (14). The expression of PSMA in tumors is, however, absent in 15–20% of men diagnosed with castration-resistant prostate cancer (CRPC), but the precise mechanisms behind this phenomenon are still unclear (15). PSMA PET has evolved as an imaging tool capable of driving more accurate and targeted approaches to PCa management. Recently, Weiner et al. detailed the historical development and contemporary impact of PSMA PET in PCa care, highlighting the advancements made and promising future directions which will be guided by clinical trials (16). In a pooled analysis of multiple prospective studies, Kawada et al. (17) showed PSMA PET increased sensitivity for detecting csPCa from 84 to 91% compared to MRI alone. Prior work has shown that PSMA PET is better able to detect more PCa in patients with biochemical recurrence compared to Choline- or Fluciclovine-based PET after primary radiation or surgery (18, 19). Specifically, when the PSA is ≤0.5 ng/mL in these patients, the detection rate is only 12.5% for Choline-based PET and 50% for PSMA PET. In a similarly designed prospective study of patients with PSA 0.2-2.0 ng/mL following surgery for PCa, the detection rate for Fluciclovine-based PET was 26%, while PSMA-based PET detected PCa in 56% (18). Additional phase III trials are required to further investigate whether PSMA PET imaging can effectively guide patients in avoiding unnecessary prostate biopsies.

Recent years have witnessed substantial progress in leveraging artificial intelligence (AI) and computer-aided diagnosis to enhance the diagnosis of PCa, encompassing both radiological and histological domains (**Figure 1**). These AI-based tools have demonstrated potential in enhancing the efficiency and precision of radiologists by streamlining or enhancing human workflow. Likewise, longstanding challenges in PCa histopathology, such as limited interobserver and intraobserver agreement in measurements and Gleason grading, are being addressed through the integration of these innovative techniques (20–23).

**Figure 1:**
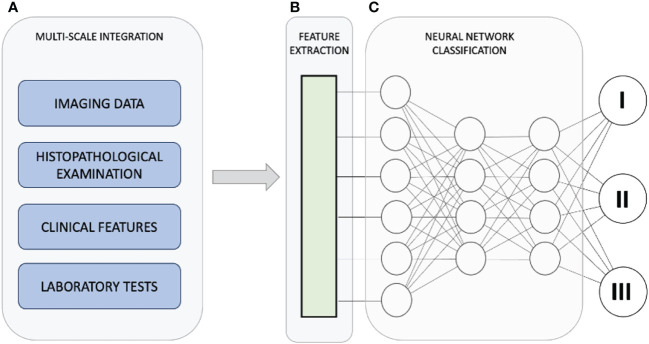
The diagnostic pathway for PCa strives to achieve timely identification of clinically significant PCa while minimizing the detection of insignificant cases. This approach seeks to strike a balance between diagnostic accuracy and the potential burden on individuals and healthcare providers. Utilizing artificial intelligence, we integrate data from various scales **(A)** to enhance the diagnosis and treatment of prostate cancer. Within the collected dataset, the AI-driven system identifies and extrapolates the features and data points most pertinent to generating the ultimate outcome **(B)**. The integration of diverse data and features culminates in a refined categorization of the condition **(C)**, emphasizing that integration involves intricate connections among diverse characteristics observed across multiple scales, rather than a mere summation of individual outcomes.

This Research Topic has provided an open discussion of how PET/CT and MRI impact the diagnosis of PCa. Liu et al. evaluated the feasibility and diagnostic performance of PSMA based 18F-DCFPyL PET/CT-ultrasound (PET/CT-US) or PET/MRI-ultrasound (PET/MRI-US) fusion targeted biopsy for intra-prostatic PET-positive lesions. From April 2018 to November 2019, they prospectively enrolled 55 subjects to perform PET/CT-US or PET/MRI-US fusion targeted biopsies for solitary PET-positive prostate lesions (two to four cores/lesion). The positive rates of PCa based on patients and biopsy cores were calculated respectively. With reference to the pathological results of biopsy cores, the MR signal characteristics in the area of the PET-positive lesion were analyzed for the patients who underwent PET/MRI. One hundred forty-six biopsy cores (82.0%) from 51 (92.7%) patients were positive for PCa; 47 (85.5%) were positive for csPCa. It is noteworthy that nine patients underwent both 18F-DCFPyL PET/CT and PET/MRI examinations; the seven patients with PCa showed abnormal MR signal in the area of the PET-positive lesion while the other two patients with prostatic hyperplasia and prostatitis showed normal MR signal in the area of the PET-positive lesion. This study indicated that 18F-DCFPyL PET/CT-US or PET/MRI-US fusion targeted prostate biopsies may be valuable for PCa diagnosis and have a high detection rate of clinically significant PCa for PET-positive lesions. PET/MR can rule out some false PET-positive lesions, which may potentially reduce unnecessary prostate biopsies.

In a recent meta-analysis involving patients with non-small cell lung cancer, there was no discernible contrast between whole-body magnetic resonance imaging (WBMRI) and PET/CT. However, such a comparative study is lacking in the context of PCa. Zhan et al. undertook a comparison between WBMRI and PET/CT for detecting bone metastases in PCa patients. Their analysis encompassed four prospective and one retrospective study, involving a total of 657 patients. Significant disparities were noted between WBMRI and PET/CT concerning sensitivity and negative likelihood ratios, whereas no notable differences were observed for specificity and positive likelihood ratios. The diagnostic odds ratio for WBMRI was found to be similar to that of PET/CT. PET/CT demonstrated higher sensitivity and negative likelihood ratios in detecting bone metastases from PCa compared to WBMRI, while no significant distinctions were detected for specificity and positive likelihood ratios. Walter et al. conducted an assessment of the MonaLisa prostate biopsy system focusing on safety, tolerability, and patient-related outcomes. This prospective study involved 228 patients who underwent Robotic-assisted transperineal MRI-US-fusion guided biopsy of the prostate. The study evaluated peri-operative side effects, functional outcomes, and patient satisfaction. On the day of biopsy, the mean pain score was 1.3 points on the Visual Analog Scale (VAS), which remained consistent on the following day. Overall, 14% of patients experienced grade I complications according to the Clavien-Dindo classification, with no higher-grade complications reported. Gross hematuria, hematospermia, and acute urinary retention occurred in 63.6%, 43%, and 14% of patients, respectively, while only one patient developed urinary tract infection. The authors concluded that robotic-assisted transperineal MRI-US-fusion guided biopsy of the prostate, performed under general anesthesia, is a safe and well-tolerated procedure. They attribute this favorable risk profile and tolerability to the minimally invasive approach involving two entry points, which allows for the omission of perioperative prophylaxis while minimizing the risk of infectious complications. In their manuscript, Gaudiano et al. provided an overview of both the common and rare features of different types of granulomatous prostatitis (GP) on mpMRI through a comprehensive literature review. Their aim was to identify radiological criteria for distinguishing this inflammatory condition from PCa and reducing the need for TRUS prostate biopsy whenever possible. Specifically, they focused on evaluating GP features within the multiparametric study protocol, which includes T2-weighted (T2w) imaging, diffusion-weighted imaging (DWI) with apparent diffusion coefficient (ADC) mapping, and dynamic contrast-enhanced (DCE) sequences. The primary limitation they encountered was the scarcity of large-scale studies on this topic due to the rarity of the disease. Consequently, the literature predominantly comprises case reports and small case series, which precluded a detailed statistical analysis. Through their literature review, they concluded that mpMRI of the prostate could be instrumental in distinguishing Bacille Calmette-Guérin (BCG)-induced GP from PCa, particularly by accurately assessing the characteristic “ring enhancement” of prostate lesions on multiphase contrast-enhanced MRI within a specific clinical context. They suggested that an mpMRI follow-up of prostatic lesions could be safely conducted in such cases. However, they noted that differentiating other types of non-necrotic GP, such as nonspecific granulomatous prostatitis, xanthogranulomatous prostatitis, and diffuse or nodular BCG-induced GP, based solely on mpMRI features, including PSA values, was not feasible. In these instances, a targeted biopsy remained the necessary approach for accurate diagnosis.

In their study, Wenhao et al. assessed the utility of quantitative T2 star (T2*) values derived from T2* mapping sequences in mpMRI for diagnosing and grading PI-RADS 3 PCa. They prospectively enrolled patients with PCa or benign prostatic hyperplasia (BPH) and collected imaging indicators, including T2* values and ADC values. Additionally, they measured levels of proteins involved in iron metabolism using enzyme-linked immunosorbent assays. Their findings revealed significant differences in three iron metabolism indexes - ferritin, hepcidin, and ferric ion (Fe) - as well as T2* values between the PCa and BPH groups and between the low ISUP group (ISUP ≤ 2) and the high ISUP group (ISUP > 2). Moreover, a significant correlation was observed between the levels of these three indicators and T2* values. Further receiver operating characteristic analysis demonstrated that the levels of iron metabolism-related indexes and T2* values exhibited strong performance in diagnosing and grading PCa. Their study highlighted the potential of T2* values in detecting and predicting the grade of PCa, as they reflect the tumor’s iron metabolism. This finding suggests that T2* values could serve as a valuable tool in the future for diagnosing and grading PCa, providing a foundation for improved clinical decision-making in PCa management. Zhang et al. conducted a comparative analysis between 99mTc-PSMA single-photon emission computed tomography (SPECT)/CT and mpMRI in detecting primary PCa. Their prospective study involved fifty-six men with suspected PCa, categorized into high- (Gleason score > 7), intermediate- (Gleason score = 7), and low-risk groups (Gleason score < 7). All patients underwent both 99mTc-PSMA SPECT/CT and mpMRI within an average interval of 3 days. They utilized maximum standardized uptake value (SUV_max_), minimum ADC_min_, and their ratio (SUV_max_/ADC_min_) as imaging parameters to differentiate between benign and malignant prostatic lesions. Their findings indicated that 99mTc-PSMA SPECT/CT and mpMRI exhibited comparable performance in detecting primary PCa, with sensitivities of 97.7% and 90.9%, specificities of 75.0% and 75.0%, and areas under the curve (AUC) of 97.4% and 95.1%, respectively. Moreover, the AUC of SUV_max_/ADC_min_ surpassed those of SUV_max_ or ADC_min_ alone. The authors identified a threshold of >7.0×10^3^ for SUV_max_/ADC_min_ in prostatic lesions, indicating a higher likelihood of malignancy. Additionally, when SUV_max_/ADC_min_ exceeded >27.0×10^3^, patients with PCa might exhibit lymph node and bone metastases. SUV_max_ exhibited a positive correlation with the Gleason score, while ADC_min_ displayed a negative correlation. SUV_max_/ADC_min_ showed a positive correlation with the Gleason score and emerged as the primary predictor of the high-risk group. The combination of 99mTc-PSMA SPECT/CT and mpMRI yielded improved diagnostic efficacy for PCa compared to either modality alone. Notably, SUV_max_/ADC_min_ emerged as a valuable differential diagnostic imaging parameter in this context.

Huang et al. conducted a comprehensive meta-analysis and systematic review to compare the diagnostic effectiveness of 68Ga-PSMA-11 PET/CT and 68Ga-PSMA-11 PET/MRI in patients with biochemically recurrent PCa after radical prostatectomy and hybrid radiotherapy and radical prostatectomy. Their analysis included studies evaluating the utility of 68Ga-PSMA-11 PET/CT or PET/MRI as a screening tool for detecting biochemically recurrent PCa. A total of 37 studies involving 8409 patients were scrutinized. To assess heterogeneity, the I^2^ statistic was employed, with the random effect model used in cases of substantial heterogeneity (I^2^ > 50%) and the fixed model in other instances. Additionally, the authors evaluated the quality of the included studies using the Quality Assessment of Diagnostic Accuracy Studies 2 (QUADAS-2) method. The combined total detection rates for 68Ga-PSMA-11 PET/CT and PET/MRI were 0.70 (95% CI: 0.65-0.75) and 0.71 (95% CI: 0.67-0.75), respectively. The authors found no significant difference in the overall detection rates for biochemical relapse between 68Ga-PSMA-11 PET/CT and PET/MRI. Moreover, the detection rates were unaffected by PSA values. Their analysis suggests that the diagnostic efficacy of 68Ga-PSMA-11 PET/CT is comparable to that of 68Ga-PSMA-11 PET/MRI in detecting biochemically recurrent PCa. However, they cautioned that not all studies employed pathological biopsies as the gold standard, highlighting the need for additional larger prospective studies to address this limitation.

In 2023, Mehmood et al. addressed the challenges posed by high-resolution and multiresolution MRI in PCa diagnosis by leveraging computer-aided diagnostic (CAD) methods. With the rapid advancement of medical technology, deep learning methods have gained prominence in this domain. These techniques not only improve diagnostic efficiency but also mitigate observer variability, consistently surpassing traditional approaches. However, resource constraints remain a significant hurdle in distinguishing aggressive from non-aggressive cancers in PCa treatment. Their study aimed to harness MRI images for PCa identification by integrating deep learning and transfer learning. They explored various convolutional neural network (CNN)-based deep learning methods for classifying PCa-related MRI images. In their approach, they developed a method for PCa classification using transfer learning on a limited image dataset to achieve high performance, aiding radiologists in prompt PCa identification. Their proposed methodology utilized the EfficientNet architecture pre-trained on the ImageNet dataset, incorporating three branches for feature extraction from different MRI sequences. The fusion of these extracted features significantly enhanced the model’s ability to accurately distinguish MRI images. Their model achieved a notable accuracy rate of 88.89% in classifying PCa. Comparative analysis revealed that their approach outperformed both traditional hand-crafted feature techniques and existing deep learning methods in PCa classification. This underscores the efficacy of their methodology in learning distinctive features from prostate images and accurately identifying cancerous regions.

Zhao et al. aimed to develop a robust model for predicting csPCa (pathological grade group ≥ 2) in PI-RADS 3 lesions within the transition zone by comparing the performance of combination models. Their study involved 243 men who underwent 3-Tesla MRI and ultrasound-guided transrectal biopsy, divided into a training cohort of 170 patients and a separate testing cohort of 73 patients. Manual segmentation of T2-weighted imaging (T2WI) and diffusion-weighted imaging (DWI) images was performed for PI-RADS 3 lesions to extract mean apparent diffusion coefficient (ADC) values and conduct radiomic analysis. Predictive clinical factors were identified using both univariate and multivariate logistic models, and the least absolute shrinkage and selection operator (LASSO) regression models were employed for feature selection and constructing radiomic signatures. The authors developed nine models combining clinical factors, radiological features, and radiomics, utilizing logistic and XGboost methods. The performance of these models was evaluated using ROC analysis and the Delong test. Among the 243 participants with a median age of 70 years, 30 were diagnosed with csPCa, leaving 213 without a csPCa diagnosis. PSA density (PSAD) emerged as the sole significant clinical factor, identified through univariate and multivariate logistic models. Seven radiomic features correlated with csPCa prediction. The XGboost model exhibited superior performance compared to eight other models (AUC of the training cohort: 0.949, and validation cohort: 0.913). However, it did not surpass the PSAD+MADC model in both the training and testing cohorts. Their findings demonstrated that the machine learning XGboost model performed best in predicting csPCa in PI-RADS 3 lesions within the transitional zone. However, the addition of radiomic classifiers did not significantly enhance the performance over the compound model of clinical and radiological findings. Thus, the Mean ADC+PSAD model was deemed the most effective and generalizable option for quantitative prostate evaluation.

Zhou et al. developed an artificial intelligence (AI)-based model for predicting the progression of castration-resistant prostate cancer (CRPC) by integrating multimodal data. They retrospectively collected data from 399 patients diagnosed with PCa across three medical centers. Regions of interest (ROIs) were delineated from three MRI sequences, namely T2WI, diffusion-weighted imaging (DWI), and ADC, and the largest section of each ROI was extracted using a cropping tool. Representative pathological hematoxylin and eosin-stained slides were selected for training a deep-learning model. Subsequently, a joint combined model nomogram was constructed. The predictive performance and goodness of fit of the model were evaluated using ROC curves and calibration curves. Decision curve analysis curves and Kaplan-Meier survival curves were generated to assess the clinical net benefit of the model and its association with progression-free survival. The AUC of the machine learning (ML) model was determined to be 0.755. The best-performing deep learning model for radiomics and pathomics was identified as the ResNet-50 model, achieving AUC values of 0.768 and 0.752, respectively. The nomogram graph illustrated that the DL model contributed the most, resulting in an AUC of 0.86 for the combined model. Calibration curves and DCA indicated that the combined model exhibited good calibration ability and provided a net clinical benefit. Additionally, the KM curve suggested that the model integrating multimodal data could guide patient prognosis and management strategies effectively. Overall, the integration of multimodal data significantly enhanced the prediction of PCa progression to CRPC. Abrahamsen et al. introduced a novel method of accounting for bone in pelvic PET/MR AC by directly predicting the errors in the PET image space caused by the lack of bone in four-class Dixon-based attenuation correction. A CNN was trained to predict the four-class AC error map relative to CT-based attenuation correction. Dixon MR images and the four-class attenuation correction µ-map were used as input to the models. CT and PET/MR examinations for 22 patients ([18F] FDG) were used for training and validation, and 17 patients were used for testing (6 [18F] PSMA-1007 and 11 [68Ga] Ga-PSMA-11). A quantitative analysis of PSMA uptake using voxel- and lesion-based error metrics was subsequently used to assess performance. In the voxel-based analysis, the proposed model reduced the median root mean squared percentage error from 12.1% and 8.6% for the four- and five-class Dixon-based AC methods, respectively, to 6.2%. The median absolute percentage error in the maximum standardized uptake value (SUV_max_) in bone lesions improved from 20.0% and 7.0% for four- and five-class Dixon-based AC methods to 3.8%. The proposed method reduces the voxel-based error and SUV_max_ errors in bone lesions when compared to the four- and five-class Dixon-based AC models. It is indubitable that MRI and PCa-specific PET represent two widely applicable, rapidly developing technologies that are becoming increasingly important to PCa diagnosis and management. While the adoption of these techniques will help us make the most informed decisions with patients, it is important to recognize that the clinical benefits and cost-effectiveness of their use are still being evaluated and debated. It should also underline that consistent image interpretation is crucial for ensuring comparable data across different clinical trials and for effectively translating research findings into routine clinical practice (24).

A significant increase in the annual number of new PCa cases is expected, with cases projected to rise from 1.4 million in 2020 to 2.9 million by 2040 (25). This rise is attributed to shifting age demographics and improvements in life expectancy, particularly driving increases in the disease burden. Late diagnosis of PCa is a widespread issue globally, with low- and middle-income countries (LMICs) particularly affected, where late diagnosis is common. To mitigate the adverse impact of this upward trend, urgent establishment of systems for earlier diagnosis in LMICs is imperative. Trials of screening are urgently required in these regions to provide valuable insights into improving early diagnosis strategies. Early diagnosis systems must incorporate innovative combinations of personnel and harness the growing capabilities of AI-based algorithms to assist in the interpretation of scans and biopsy samples. This multifaceted approach is essential to address the challenges posed by rising case numbers and improve outcomes for individuals affected by prostate cancer worldwide. Tackling the intricate dimensions of PCa, both in its temporal progression and spatial manifestations, holds the promise of uncovering deeper insights into its origins and evolution (26). This comprehensive approach stands to provide a more cohesive conceptual framework, enhance the interpretation of experimental findings, guide targeted research endeavors, and offer a systematic way to organize the vast array of existing knowledge by identifying commonalities or shared characteristics among different types of tumors. Encouragingly, collaboration among experts from diverse disciplines such as engineering, clinical medicine, biology, and mathematics continues to drive forward efforts towards achieving a unified and quantifiable understanding of cancer’s complexities. Crucially, technology plays an indispensable role in this pursuit, serving as a vital catalyst for scientific progress.

## Author contributions

FG: Writing – review & editing, Writing – original draft, Supervision, Conceptualization. GT: Writing – review & editing, Writing – original draft, Supervision, Conceptualization.
